# Novel Insights in the Regulatory Mechanisms of Ferroptosis in Hepatocellular Carcinoma

**DOI:** 10.3389/fcell.2022.873029

**Published:** 2022-05-19

**Authors:** Shiwen Ma, Yao Mawulikplimi Adzavon, Xiaohu Wen, Pengxiang Zhao, Fei Xie, Mengyu Liu, Xuemei Ma

**Affiliations:** ^1^ Faculty of Environment and Life, Beijing University of Technology, Beijing, China; ^2^ Beijing Molecular Hydrogen Research Center, Beijing, China

**Keywords:** ferroptosis, iron, lipid peroxidation, hepatocellular carcinoma, iron homeostasis

## Abstract

Ferroptosis is a newly defined programmed cell death, which by its mechanism differs from other programmed cell death processes such as apoptosis, necrosis, and autophagy. It has a unique morphology and biological properties that antioxidants and iron-chelating agents can regulate. Ferroptosis has the characteristics of iron ion deposition and dependence on lipid peroxidation. It can affect the progression of many cancers, including liver cancer, by inducing an intracellular iron-dependent accumulation of reactive oxygen species, providing new possibilities for cancer treatment. At present, great progress has been made in exploring the molecular mechanism of ferroptosis. In this review, we summarize the characteristics, mechanisms, and regulatory factors of ferroptosis in detail, discuss the progress of ferroptosis research in liver cancer, and provide directions and new ideas for the treatment of hepatocellular carcinoma.

## 1 Introduction

Regulatory cell death (RCD) is a common process in organisms, essential to restoring tissue homeostasis or biological balance after stress. RCD is defined as a death process dependent on specific molecular mechanisms, which can be regulated through specific pharmacological and genetic interventions to promote the selective removal of harmful cells or activate specific pathological states (Del Re et al., 2019). RCD occurs as a homeostatic mechanism during development and aging, but could also originate from disturbances in the intracellular or extracellular microenvironment (Thompson, 1995). In addition to RCD, other forms of death programs have been described (Del Re et al., 2019). In 2003, Dolma et al. discovered a nonapoptotic form of cell death induced by Erastin in tumors with RAS mutations (Dolma et al., 2003). Later in 2008, they identified two additional compounds, RSL3 and RSL5, with the same nonapoptotic cell death-inducing potential as Erastin (Yang and Stockwell, 2008). The newly discovered death program was later identified in 2012 as an intracellular iron-dependent form of cell death caused by cellular accumulation of lipid peroxides and was called ferroptosis (Dixon et al., 2012). Ferroptosis cells have unique morphological and bioenergy characteristics that differentiate them from other forms of regulated cell death such as apoptosis and necrosis (Dixon et al., 2012; Bersuker et al., 2019; Xia et al., 2019). At the subcellular level, mitochondria in ferroptosis cells are smaller, have a higher membrane density, have cristae that shrink or disappear and show rupture of the outer mitochondrial membrane (Dixon et al., 2012; Friedmann Angeli et al., 2014; Hassannia et al., 2019). Ferroptosis can be induced by various types of small molecules (Table 1), including Erastin and derivatives, sulfasalazine (SAS), glutamate, and drugs such as Sorafenib, cisplatin, artemisinin, and lanperisone (Stockwell et al., 2017; Yu et al., 2017). These molecules act on the system Xc- and reduce intracellular glutathione content resulting in a cellular redox imbalance (Gout et al., 2001; Yagoda et al., 2007; Dixon et al., 2012). Other inducers, including RAS selective lethal compound 3 (RSL3), DPI2, DPI7, directly inhibit glutathione peroxidase 4 (GPX4), resulting in accumulation of lipid peroxides (Yang et al., 2014). Furthermore, the glutathione synthesis interrupter butylthionyl sulfoxide (BSO) can also induce ferroptosis (Griffith, 1982; Shaw et al., 2011; Dixon et al., 2012; Dixon et al., 2014; Eling et al., 2015; Louandre et al., 2015; Lőrincz et al., 2015; Sun et al., 2020). Since the increase in reactive oxygen species (ROS) and iron accumulation are the two most important factors in the ferroptosis process, antioxidants such as ferrostatins-1 and liproxstatins, exogenous iron chelating agents such as DFO (Dixon et al., 2012; Friedmann Angeli et al., 2014; Yang et al., 2014; Chen et al., 2021), and endogenous compounds such as glutathione, ubiquinone, vitamin E and selenium (Dixon et al., 2012; Homma and Fujii, 2015; Manz et al., 2016; Stockwell et al., 2017) can be used as inhibitors of ferroptosis.

**TABLE 1:** Compounds that modulate ferroptosis.

Drugs	Targets	Modulators	Impact on ferroptosis	References
Erastin	System Xc-;VDACs	Inducer	Inhibits the entry of cystine, causes glutathione depletion; combines with VDACs on the outer mitochondrial membrane, causes mitochondrial metabolism disorder and dysfunction	(Yagoda et al., 2007; Dixon et al., 2012)
sulfasalazine	System Xc-	Inducer	It inhibits the entry of cystine, causes glutathione depletion	Gout et al. (2001)
Sorafenib	System Xc-	Inducer	It inhibits the entry of cystine, causes glutathione depletion	(Dixon et al., 2012; Dixon et al., 2014; Louandre et al., 2015)
glutamate	System Xc-	Inducer	High extracellular glutamate concentrations prevent cystine import, causes glutathione depletion	Dixon et al. (2012)
lanperisone	Unknown	Inducer	Reduce glutathione	Sun et al. (2020)
artemisinin	Fe^2+^	Inducer	Promotes the phagocytosis of ferritin to increase the level of free iron in cells	Eling et al. (2015)
BSO	GSH	Inducer	Inhibits GSH synthesis, causes the decreased activity of GPX4	Griffith, (1982)
cisplatin	GSH	Inducer	Combines with GSH to form a Pt-GS complex, causes the loss of GSH and decreases the activity of GPX4	Lőrincz et al. (2015)
DPI2	GSH	Inducer	Inhibits GSH synthesis, causes the decreased activity of GPX4	Yang et al. (2014)
statins	HMGCR	Inducer	Inhibits the biosynthesis of selenoprotein (such as GPX4) and coenzyme Q10	Chen et al. (2021)
RSL3	GPX4	Inducer	Directly binds to GPX4 protein, causing its inactivation	Yang et al. (2014)
deferoxamine	Fe^2+^	Inhibitor	Iron chelator, depletes iron	(Dixon et al., 2012; Chen et al., 2021)
ferrostatin-1	lipid peroxidation	Inhibitor	Antioxidant, blocks lipid peroxidation	Dixon et al. (2012)
liproxstatin-1	lipid peroxidation	Inhibitor	Antioxidant, blocks lipid peroxidation	Yang et al. (2014)
ubiquinone	lipid peroxidation	Inhibitor	Antioxidant, blocks lipid peroxidation	Friedmann Angeli et al. (2014)
vitamin E	lipid peroxidation	Inhibitor	Antioxidant, blocks lipid peroxidation	Manz et al. (2016)
glutathione	glutaminolysis	Inhibitor	Unknown	Homma and Fujii, (2015)
selenium	selenoproteins	Inhibitor	Increases abundance of selenoproteins	(Dixon et al., 2012; Stockwell et al., 2017)

Ferroptosis is associated with various physiological and pathological processes (Jenkins et al., 2020; Shen et al., 2020; Jiang et al., 2021), and its pathophysiological relevance has been well documented in a growing number of diseases such as neurodegeneration (Devos et al., 2014; Chen et al., 2015; Hambright et al., 2017), fibrosis (Zhang et al., 2018; Yu et al., 2020), autoimmune (Hu et al., 2019; Kim et al., 2019) and pulmonary diseases (Park et al., 2019). Ferroptosis has also been proven to be very important for various tumors, including hepatocellular carcinoma, lung cell carcinoma, lymphoma, pancreatic ductal cell carcinoma, and renal cell carcinoma (Louandre et al., 2013; Yang et al., 2016; Chen et al., 2021; Li et al., 2021). In this review, we focus on research progress on ferroptosis in liver cancers and provide the latest information on the basic mechanisms that contribute to the regulation of ferroptosis in this cancer.

## 2 General Overview of the Mechanisms of Ferroptosis

**FIGURE 1:**
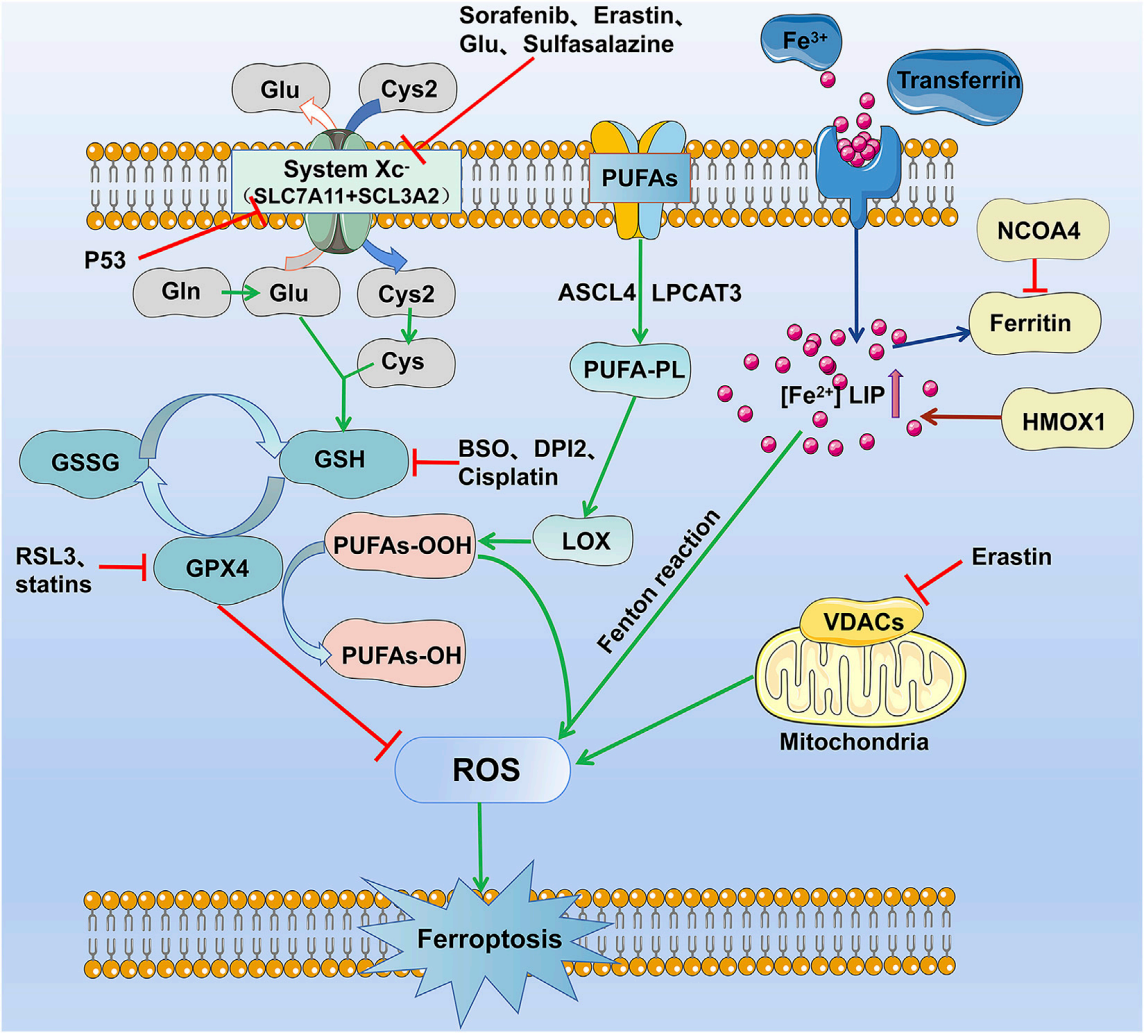
The occurrence and regulation mechanism of ferroptosis in cells. The figure highlights the five currently known mechanisms involved in ferroptosis: lipid reactive oxygen metabolism pathway, cystine glutamate transport receptor (System Xc-) metabolic pathway, iron metabolism pathway, and VDAC receptor pathway.

Induction of ferroptosis required iron and iron-dependent peroxidation enzymes (Yang and Stockwell, 2008; Wenzel et al., 2017), phospholipids with polyunsaturated fatty acids, and inhibition of the pathways involved in the reparation of lipid peroxidation. The contribution of these three hallmarks to ferroptosis has been extensively investigated, and current knowledge of their mechanism in the process is reviewed and summarized in Figure 1 and Table 2.

**TABLE 2:** The regulatory mechanisms of ferroptosis.

Mechanism	Target	Proposed Mechanism	References
Iron homeostasis	Transferrin↑ Ferroportin↓	LIP provides iron by TFR-mediated endocytosis or ferritin degradation and participates in Fenton reaction to further promote lipid peroxidation	Gaschler and Stockwell, (2017)
Inhibition of system Xc-	Depletion of cysteine	Decreases glutathione levels, impaires glutathione peroxidase 4 (GPX4) activity, ROS accumulation, and subsequent lipid peroxidation	Badgley et al. (2020)
Lipid peroxidation	Enzymatic reactions	Mediated by the activity control of LOXs and COXs	Conrad and Pratt, (2019)
	Nonenzymatic reactions	A free radical-driven chain reaction in which reactive oxygen species (ROS) trigger polyunsaturated fatty acid oxidation	Gaschler and Stockwell, (2017)
GPX4 and GPX4-independent	GPX4 inactivation/depletion	Reduces reactive phospholipid hydroperoxides (PL-OOH) to nonreactive phospholipid alcohols (PL-OH), interrupts free radical chain reactions, inhibits lipid peroxidation, and suppress ferroptosis	Maiorino et al. (2018)
	Ferroptosis suppressor protein 1 (FSP1)	Transports and folds mitochondrial intermembrane proteins, protects cells from ferroptosis induced by inhibition or genetic deletion of GPX4	Bersuker et al. (2019)
	Voltage-dependent anion channel (VDAC)	Erastin combines with VDAC2 and VDAC3 in the outer mitochondrial membrane to change membrane permeability, slow the oxidation of NADH, and change the ion selectivity of the channel, allowing only cations to enter mitochondria	Yagoda et al. (2007)
	Protein Kinases	Ferroptosis involves multiple signaling pathways that can dictate cell susceptibility to ferroptosis under specific biological conditions	Zhang et al. (2021)
	Cell cycle regulators	p53 controls ferroptosis through complex mechanisms involving transcriptional and post-transcriptional modifications	Gnanapradeepan et al. (2018)

### 2.1 Regulation of Iron Homeostasis and Implications for Ferroptosis

Iron is an important factor in the formation of free radicals and lipid peroxidation and plays a pivotal role in ferroptosis. Increased iron absorption and decreased iron output make cancer cells sensitive to oxidative damage and ferroptosis (Liang et al., 2019). Transferrin and its receptors transport iron into the cell and store it in the form of ferritin, while intracellular iron is exported through ferroportin to maintain iron balance in the cell (De Domenico et al., 2008; Trujillo-Alonso et al., 2019). The labile iron pool (LIP) exists mainly in the cytoplasm in the form of Fe^2+^, which can directly catalyze the formation of hydroxyl radicals with strong activity through the Fenton reaction and further promote lipid peroxidation (Gaschler and Stockwell, 2017; Yan et al., 2021). In eukaryotes and most prokaryotes, iron participates in the synthesis of iron-sulfur clusters (Fe-S), heme, and other cofactors. It is mainly involved in energy metabolism, oxygen transport and metabolism, cell respiration and electron transfer, signal transduction, central nervous system myelin, various neurotransmitter formation, DNA replication and repair, enzyme reaction, and other important physiological processes (Beard, 2001; Cronin et al., 2019; Braymer et al., 2021). Furthermore, iron and its derivatives, such as heme or iron-sulfur [Fe-S] clusters, can affect the activity of enzymes that catalyze ROS production, such as NADPH oxidases (NOX), lipoxygenases (LOX), and mitochondrial electron transport complexes, which stimulate ROS production and thus lead to ferroptosis (Wang et al., 2020). In addition to its role in ferroptosis, iron is an essential trace element for normal body functioning. Iron regulates different biological processes, including the cellular metabolism of proteins and enzymes, and defects in maintaining its cellular homeostasis by iron overload or iron deficiency could lead to human disorders (De Domenico et al., 2008; Ng et al., 2020).

The cytosolic iron pool can be affected in various ways. For example, inhibition of nitrogen fastening 1 (NFS1), a cysteine desulfurase, sensitizes cells to ferroptosis by increasing the transferrin receptor (TFRC) and decreasing ferritin levels (FTH) by decompressing cysteine sulfur to produce a group of iron-sulfur (Alvarez et al., 2017). The lysosomal degradation of ferritin can lead to a large amount of cytosolic iron accumulation. This process involves Nuclear Receptor Coactivator 4 (NCOA4), which can bind and transport ferritin to autophagosomes where ferritin is degraded and lysosomal iron exported in the cytosol. Therefore, inhibition of lysosomal activity or silencing of NCOA4 can inhibit ferroptosis (Mancias et al., 2014; Quiles del Rey and Mancias, 2019; Mou et al., 2021). HMOX1 catalyzes the degradation of heme to Fe^2+^, biliverdin, and carbon monoxide, enhancing ferroptosis by increasing LIP (Kwon et al., 2015; Chang et al., 2018; Conrad and Pratt, 2019). Besides, HMOX1 can also impact cell protection through its antioxidant activity (Sun et al., 2016; Conrad and Pratt, 2019). The circulating peptide hormone Hepcidin, a beta-defensin-like peptide coded by the HAMP gene and secreted primary by hepatocytes, is a principal regulator of systemic iron homeostasis (Verga Falzacappa and Muckenthaler, 2005; De Domenico et al., 2008; Yang et al., 2020). Hepcidin acts as a negative regulator of iron transport into plasma by binding to the ferroportin, causing its internalization and lysosomal degradation (Nemeth et al., 2004; De Domenico et al., 2007), leading to an increase in the level of cytosolic iron that explains the contribution of hepcidin to ferroptosis regulation (Yang et al., 2020).

### 2.2 System X_C_^−^ and Ferroptosis

Cysteine/Glutamate antiporter referred to as System X_C_^−^ is a heterodimer composed of SLC7A11 and SLC3A2, a sulfide-linked system, and an important target for the induction of ferroptosis. System X_C_^−^ facilitates the import of cystine and glutamate export in a 1:1 ratio. Cystine that enters the cell via the antiporter is reduced to cysteine and used in the biosynthesis of reduced glutathione (GSH) (Jiang et al., 2021). GSH is a tripeptide of glutamate, cysteine, and glycine, synthesized by the consecutive action of the cytoplasmic enzymes glutamate-cysteine ligase (GCL) and glutathione synthase (GSS), respectively (Meister, 1995). GSH is the most abundant antioxidant in mammalian cells, and it prevents the cellular accumulation of reactive oxygen species (ROS). Therefore, depletion of cysteine by deletion of SLC7A11 or through inhibition of the system Xc^−^ using chemical probes such as Erastin or Sorafenib results in a decrease in the level of GSH, impaired glutathione peroxidase 4 (GPX4) activity, accumulation of ROS, and subsequent lipid peroxidation, required for the execution of ferroptosis-mediated cell death (Yang et al., 2014; Badgley et al., 2020; Stockwell and Jiang, 2020).

### 2.3 Lipid Peroxidation in Ferroptosis

Lipid peroxidation is the hallmark of ferroptosis. It is a complex biological process of oxidative degradation of lipids observed in plants and animals. It is a chain of enzymatic and nonenzymatic reactions initiated by hydrogen abstraction or the addition of oxygen radicals, resulting in the oxidative damage of polyunsaturated fatty acids (PUFA) (Yin et al., 2011). Nonenzymatic lipid peroxidation is a free radical-driven chain reaction in which reactive oxygen species (ROS) trigger polyunsaturated fatty acid oxidation (Vigor et al., 2014) and are facilitated by Fe^2+^ (Repetto et al., 2010; Gaschler and Stockwell, 2017). During this process, hydroxyl radicals extract hydrogen from polyunsaturated fatty acids to produce carbon-centered phospholipid radicals that subsequently react with oxygen to form lipid peroxide radicals (PLOO·) (Hassannia et al., 2019). The PLOO can propagate the chain reaction by extracting another hydrogen from adjacent polyunsaturated fatty acids to form lipid hydroperoxide (PLOOH) and a new lipid free radical, triggering another chain reaction of lipid peroxidation (Yin et al., 2011; Gaschler and Stockwell, 2017; Maiorino et al., 2018; Conrad and Pratt, 2019). In contrast, enzymatic lipid peroxidation is mediated in a controlled manner by the activity of LOXs (Gaschler and Stockwell, 2017; Conrad and Pratt, 2019) and cyclooxygenases (COXs) (Rouzer and Marnett, 2003). LOX, a non-heme iron dioxygenase, and COXs catalyze the dioxygenation of free and esterified PUFA to produce various lipid hydroperoxides, PLOOH. In mammalian cells, linoleic acid (LA) and arachidonic acid (AA) are the most abundant PUFA and substrates for LOX. Free PUFA can be esterified by activation of the acyl-coenzyme A synthase long-chain family member 4 (ACSL4) and bound to membrane phospholipids by lysophosphatidylcholine acyltransferase 3 (LPCAT3). ACSL4 up-regulation is considered a biomarker and contributor to ferroptosis (Doll et al., 2017; Lu et al., 2018). In the presence of ferrous iron, PLOOH can be broken down to the alkoxy lipid radical (PLO), which promotes further spread of lipid peroxidation by binding to another PUFA; on the other hand, PLOOH can break down into 4-hydroxynonenal (4-HNE) or malondialdehyde (MDA), causing the formation of adducts that disrupts the structure and/or function of proteins. Peroxidation of phospholipid and production of 4-HNE or MDA can cause membrane instability and permeability, leading to cell death. When inhibition of lipid peroxidation is out of control, this iron and oxygen catalyzed chain process leads to membrane destruction and to ferroptosis (Yin et al., 2011; Gaschler and Stockwell, 2017; Conrad and Pratt, 2019).

### 2.4 GPX4 and GPX4-Independent Regulation of Ferroptosis

Although the importance of the GSH-GPX4 axis in ferroptosis, recent work has uncovered GPX4-independent mechanisms that control ferroptosis. These mechanisms are summarized in the sections below.

#### 2.4.1 GSH-Glutathione Peroxidase 4(GPX4) Axis

GSH-GPX4 axis is considered the main system that controls ferroptosis in mammals. Glutathione peroxidase 4 (GPX4) can reduce reactive phospholipid hydroperoxides (PL-OOH) to nonreactive phospholipid alcohols (PL-OH), which can interrupt free radical chain reactions, inhibit lipid peroxidation, and thus suppress ferroptosis. GPX4 does so using its catalytic selenocysteine residue and two electrons donated by GSH or low-molecular thiols or protein thiols (Maiorino et al., 2018; Jiang et al., 2021). GSH depletion caused by cysteine deprivation directly inactivates GPX4 and leads to subsequent ferroptosis. Moreover, alteration of GPX4 activity by pharmacological inhibitors such as RSL3 or Altretamine or genetic methods leads to rapid accumulation of lipid ROS, which can cause ferroptosis (Shen et al., 2018; Weiland et al., 2019; Jiang et al., 2021).

#### 2.4.2 Ferroptosis Suppressor Protein 1 (FSP1)

FSP1, known for its role in transporting and folding mitochondrial intermembrane proteins (Reinhardt et al., 2020), was found to protect cells from ferroptosis induced by inhibition or genetic deletion of GPX4 (Bersuker et al., 2019; Doll et al., 2019). The anti-ferroptosis function of FSP1 is based on its NADH:ubiquinone oxidoreductase activity (Elguindy and Nakamaru-Ogiso, 2015), through which it suppresses lipid peroxidation by reducing ubiquinone to ubiquinol, which in turn may directly reduce lipid radicals to end lipid autoxidation or indirectly by regenerating the antioxidant, vitamin E (Elguindy and Nakamaru-Ogiso, 2015; Bersuker et al., 2019; Doll et al., 2019).

#### 2.4.3 Voltage-Dependent Anion Channel (VDAC)

The first described inducer of ferroptosis, Erastin, binds directly to two isoforms of the VDAC family, VDAC2 and VDAC3, and this interaction was required for Erastin-mediated lethality (Yagoda et al., 2007). Located in the outer mitochondrial membrane, VDAC mediates and controls the exchange of ions and metabolites between mitochondria and cytoplasm in eukaryotic cells through dynamic gating interaction. When VDAC is closed, mitochondrial transport function is restricted, metabolism is inhibited, and a low ATP/ADP ratio is maintained, thus reducing oxidative stress (Maldonado et al., 2010; Lemasters, 2017). The opening of VDAC mediates the entry of respiratory substrates, ADP, phosphoric acid, and other substances into mitochondria, leading to increased mitochondrial metabolism, reduced glycolysis, and increased ROS production (Lemasters, 2017). Erastin combines with VDAC2 and VDAC3 in the outer mitochondrial membrane to change membrane permeability, slow the oxidation of NADH, and change the ion selectivity of the channel, allowing only cations to enter mitochondria (Yagoda et al., 2007; Maldonado et al., 2013); this leads to increased ROS production and increased lipid peroxidation, which in turn causes ferroptosis (Yagoda et al., 2007; Maldonado et al., 2013).

#### 2.4.4 Protein Kinases in Ferroptosis

Ferroptosis involves multiple signaling pathways that can dictate cell susceptibility to ferroptosis under specific biological conditions. For example, the activation of the Ras-RAF-MEK-ERK pathway is necessary for Erastin-induced cell death in tumor cells harboring activating mutations in the RAS-RAF-MEK pathway (Xie et al., 2016) but not in acute myeloid leukemia where only inhibition of p38 and JNK was associated with resistance to Erastin-induced cell death (Yagoda et al., 2007; Yu et al., 2015) or in human pancreatic islet-like cells where p38 and JNK activation was necessary for Erastin induced ferroptosis to occur (Li and Leung, 2020) or to cold-induced ferroptosis in multiple cell lines (Hattori et al., 2017). A wide variety of agents activate AMP-activated protein kinase (AMPK) (Hawley et al., 2010), and the stress condition underlying this activation is determinant for the AMPK function during ferroptosis (Zhu et al., 2019; Zhao et al., 2020b; Lee et al., 2020; Yao et al., 2021). Recently, activation of the cellular energy sensor AMPK under energy stress induced by glucose depravation was found to block ferroptosis by impairing the biosynthesis of PUFAs, essential for lipid peroxidation that drives ferroptosis (Lee et al., 2020; Yao et al., 2021). The same AMPK activation, but this time under non-metabolic stress conditions, was required for ferroptosis induction in several experimental conditions (Zhu et al., 2019; Zhao et al., 2020b). In addition to MAPKs and AMPK, several other kinases have been reported as a positive or negative regulators of ferroptosis (Zhang et al., 2020a; Zhao et al., 2020a; Zhang et al., 2021; Zhong et al., 2021).

#### 2.4.5 Cell Cycle Regulators in Ferroptosis

The tumor suppressor p53, encoded by the TP53 gene, is a key regulator of cell cycle, senescence, and apoptosis and plays an important role in the occurrence and development of tumors (Liu J. et al., 2020). Beyond the functions mentioned above, p53 is believed to also control ferroptosis through complex mechanisms involving transcriptional and post-transcriptional modifications and is reviewed in detail elsewhere (Gnanapradeepan et al., 2018; Kang et al., 2019; Liu J. et al., 2020). Moreover, several direct targets of p53, including SLC7A11, GLS2, PTGS2, and SAT1, have been discovered to play a role in ferroptosis. The tumor suppressor p53 could act as an inducer or inhibitor of ferroptosis depending on cell types, energy state, and p53 status (Gnanapradeepan et al., 2018). Besides p53, the loss of function of the retinoblastoma (Rb) protein, well known for its ability to regulate the activity of the transcription factors E2F, was found to promote Sorafenib induced ferroptosis in hepatocellular carcinoma (Louandre et al., 2015). Furthermore, p21 encoded by the CDKN1A gene was a barrier to ferroptosis independent of p53 (Venkatesh et al., 2020). These data suggest a probable implication of cell cycle regulators in the ferroptosis process (Jiang et al., 2015).

### 3 Research Progress on Ferroptosis in HCC

**FIGURE 2:**
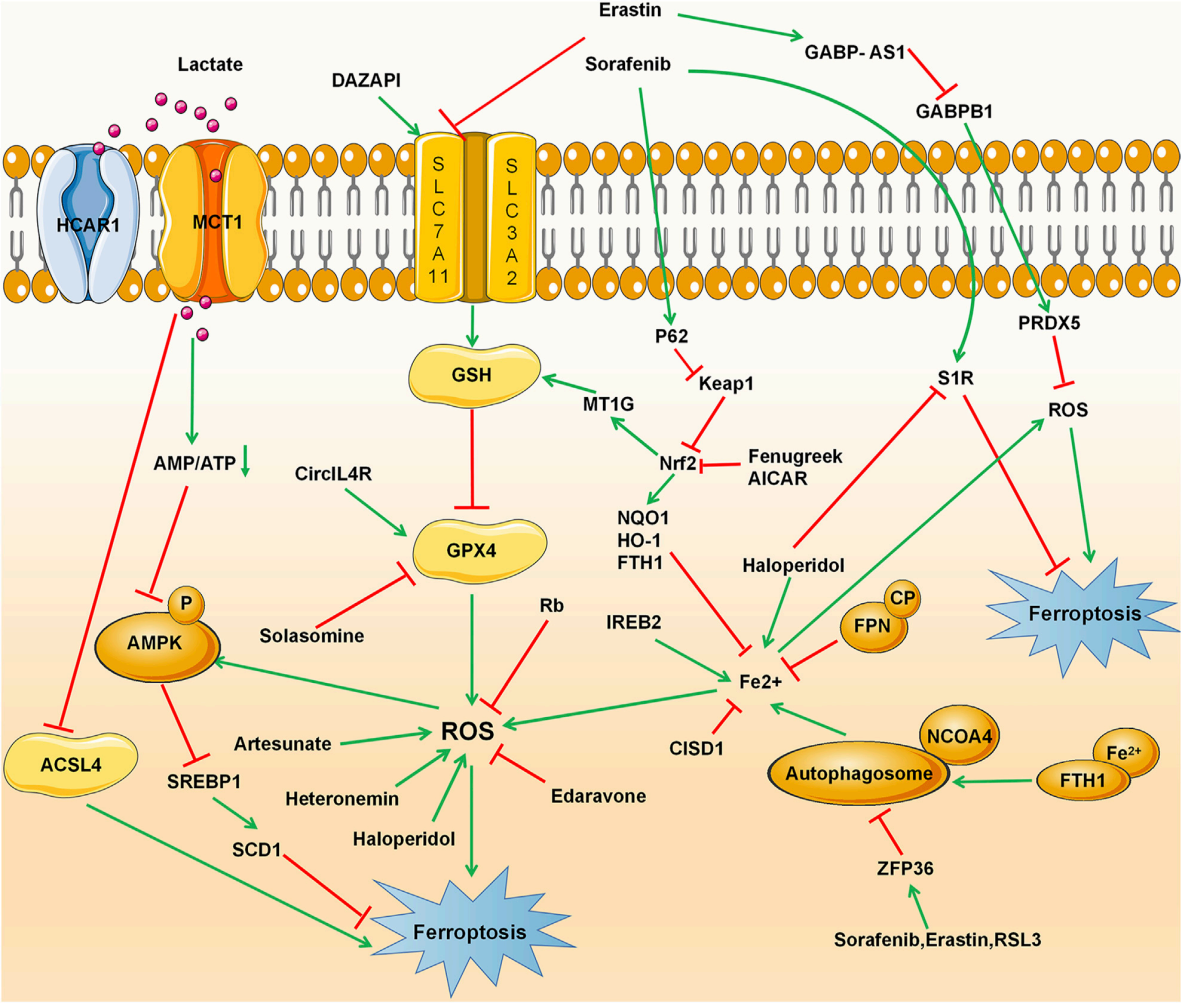
Ferroptosis signaling pathway in hepatocellular carcinoma (HCC), including conventional drivers and suppressors, non-coding RNAs, RNA-binding proteins, ACSL4, and metallothionein.

Current research has shown that ferroptosis could be induced in many cancers such as hepatocellular carcinoma, lung cell carcinoma, lymphoma, pancreatic ductal cell carcinoma, and renal cell carcinoma and could be considered a therapeutic strategy. HCC is one of the most common primary malignant tumors and the third leading cause of cancer-related death (Siegel et al., 2019). Generally, surgical resection and liver transplantation can treat liver cancer if diagnosed in its early stages. However, in advanced stages, only Sorafenib is currently approved by the FDA for advanced HCC (Roxburgh and Evans, 2008; Villanueva, 2019). Many other therapies have been tested in clinical trials for the past decades, but most of them did not receive approval for HCC patients. Even some of the approved drugs later failed to inhibit tumor growth due to the emergence of resistance mechanisms. Therefore, it is important to find new and better treatment strategies for patients with HCC, which continues to increase. Ferroptosis, which is considered the most promising tumor growth inhibitor, can affect the occurrence and development of HCC by regulating intracellular iron levels and intracellular reactive oxygen species, providing new treatment options for patients with liver cancer (Couri and Pillai, 2019). This section focuses mainly on current research progress that evaluated ferroptosis in HCC and highlights the mechanisms involved in the process (Figure 2 and Table 3).

**TABLE 3:** Regulators of ferroptosis in HCC.

Effect	Regulator	Target	References
Ferroptosis drivers	Heteronemin	Induces the formation of reactive oxygen species (ROS) and to trigger ROS removal by mitochondrial SOD2	Chang et al. (2021)
	Saponin Formosanin C	Induces higher levels of NCOA4 and lower levels of ferritin heavy chain 1 (FTH1)	Lin et al. (2020)
	Solasonine	Inhibits GPX4	Jin et al. (2020)
	Artesunate	Synergizes with sorafenib in inducing ferroptosis	Li et al. (2021)
	Quiescin sulfhydryl oxidase 1 (QSOX1)	Inhibits activation of the master antioxidant transcription factor NRF2 and proposes QSOX1	Sun et al. (2021)
	Auranofin	Synergizes with BSO inhibiting of GPX4	Yang et al. (2020)
	Haloperidol	Binds to the sigma 1 receptor (S1R), increasing cellular levels of Fe^2+^ and lipid peroxidation and decreasing the level of cellular GSH	Bai et al. (2017)
Ferroptosis suppressors	Ceruloplasmin (CP)	Regulats iron homeostasis and lipid reactive oxygen species	Shang et al. (2020)
	Lactate	Deactivates AMP-activated protein kinase (AMPK), leading to upregulation of sterol regulatory element-binding protein 1 (SREBP1) and downstream stearoyl-coenzyme A (CoA) desaturase-1 (SCD1), enhances the production of monounsaturated fatty acids with anti-ferroptosis properties	Zhao et al. (2020b)
	Sigma-1 receptor (S1R)	Regulates the accumulation of reactive oxygen species through NRF2	Bai et al. (2019)
	O-GlcNAcylated c-Jun	Controls GSH synthesis	Chen et al. (2019)
	Edaravone	Free radical scavenger	Homma et al. (2019)
Non-coding RNAs	MicroRNA-214-3p	Inhibits ATF4 in liver cancer cells, accelerates ferroptosis	Bai et al. (2020)
	ubiquitin-like modifier activating enzyme 1 (UBA1)	Modulats cell phenotypes and ferroptosis via the NRF2 pathway	Shan et al. (2020)
	GABPB1-AS1	Regulates the ferroptosis process of HCC cells caused by erastin	Qi et al. (2019)
	circIL4R	A tumor promoter and ferroptosis inhibitor in HCC by the miR-541-3p/GPX4 network	Xu et al. (2020)
	CIARS	Suppresses the inhibition of autophagy mediated by the RNA binding protein ALKBH5	Liu et al. (2020b)
RNA-binding proteins	DAZAP1	Interacts with the SLC7A11 mRNA	Wang et al. (2021)
	ZFP36	Regulate ferroptosis	Zhang et al. (2020b)
	IRP2	Regulate ferroptosis	Moroishi et al. (2011)
	ELAVL1	Up-regulation of ELAVL1 promoted the production of BECN1/Beclin1 by binding to the AU-rich elements in the 3ʹ-UTR of BECN1 mRNA, triggering autophagy activation	Zhang et al. (2018)
Ferroptosis biomarker in HCC	ACSL4	Esterifies free PUFA and binds to membrane phospholipids by LPCAT3	Feng et al. (2021)
Negative regulator of ferroptosis	Metallothionein-1G	Sorafenib enhances expression of the metal ion protein-1 (MT1) gene due to the activity of the transcription factor NRF2, which has a binding site in an antioxidant response element found in the MT-1G promoter	Sun et al. (2016)

### 3.1 Conventional Drivers and Suppressors of Ferroptosis in HCC

Currently, a large number of studies have shown that in addition to traditional ferroptosis inducers such as inhibitors of GPX4 and system Xc- (Table1), many other substances can induce and play an important role in the ferroptosis process in HCC. Some of these substances may act alone, while others must be combined with conventional ferroptosis inducers or chemotherapies. Recently, a marine terpenoid, heteronemin, has been found to inhibit HCC cell lines HA22T and HA59T through ROS-MAPK-mediated apoptosis and ferroptosis (Chang et al., 2021). At the same time, Saponin Formosanin C, a natural compound isolated from *Paris formosana Hayata,* has been found to induce ferroptosis in HepG2 cells with higher levels of NCOA4 and lower levels of ferritin heavy chain 1 (FTH1) (Lin et al., 2020). Solasonine, obtained from *Solanum melongena,* has been proposed to act as a GPX4 inhibitor that promotes HCC cell lines HepG2 and HepRG ferroptosis by destroying the glutathione peroxidase 4-induced glutathione redox system (Jin et al., 2020). In a study led by Li *et al.*, Artesunate, a clinically well-tolerated compound, synergized with sorafenib in inducing ferroptosis in HCC cell lines Huh7, SNU-449, and SNU-182 (Li et al., 2021). Sun *et al.* has shown that the combination therapy of Quiescin sulfhydryl oxidase 1 (QSOX1) and sorafenib sensitized HCC cells to oxidative stress by inhibiting activation of the master antioxidant transcription factor NRF2 and proposed QSOX1 to serve as a new therapeutic target in HCC or other types of EGFR-dependent tumors (Sun et al., 2021). Other approaches combining Auranofin and BSO or Erastin and BSO have shown a beneficial effect in Huh7 cells by promoting ferroptosis induced by inhibition of GPX4 (Lippmann et al., 2020). The psychotropic drugs haloperidol, which binds to the sigma 1 receptor (S1R), has been found to improve erastin and sorafenib-induced ferroptosis by increasing cellular levels of Fe^2+^ and lipid peroxidation and decreasing the level of cellular GSH (Bai et al., 2017).

In addition to inducing ferroptosis, preventing its inhibition could also be an alternative strategy for HCC treatment. Shang *et al.* have shown that Ceruloplasmin (CP), a copper-containing glycoprotein and member of the multicopper oxidase family (Vashchenko and MacGillivray, 2013), can suppress erastin and RSL3 by regulating iron homeostasis and lipid reactive oxygen species in HCC cell lines HepG2 and Hep3B (Shang et al., 2020). Lactate, commonly found in the microenvironment of aerobic glycolytic cancer (de la Cruz-López et al., 2019; Kim and DeBerardinis, 2019; Pérez-Tomás and Pérez-Guillén, 2020), was recently identified to enhance resistance to ferroptosis damage induced by ferroptosis inducers RSL3 and erastin when it is uptake into HCC cells through the monocarboxylate transporter 1 (MCT1). These lactate-rich cancer cells has been found to deactivate AMP-activated protein kinase (AMPK), leading to upregulation of sterol regulatory element-binding protein 1 (SREBP1) and downstream stearoyl-coenzyme A (CoA) desaturase-1 (SCD1), which enhance the production of monounsaturated fatty acids with anti-ferroptosis properties (Zhao et al., 2020b). Several other ferroptosis inhibitors have also been observed in HCC, among which the Sigma-1 receptor (S1R) that regulates the accumulation of reactive oxygen species through NRF2 (Bai et al., 2019), or the O-GlcNAcylated c-Jun that controls GSH synthesis (Chen et al., 2019) and Edaravone, a clinically approved free radical scavenger for the treatment of acute ischemic stroke and amyotrophic lateral sclerosis (Homma et al., 2019) have been showed to protect mouse hepatoma Hepa 1-6 cells from ferroptosis.

### 3.2 Other Regulators of Ferroptosis in HCC

Identifying new therapeutic targets or prognostic markers is of great significance in developing a precise and better treatment for liver cancer. In addition to the conventional ferroptosis inducers and inhibitors, several other regulators have been identified and could be considered potential targets for treating HCC patients.

#### 3.2.1 Non-Coding RNAs and RNA-Binding Proteins in Ferroptosis of HCC

MiRNAs and RNA-biding proteins are pivotal participants and regulators in the development and progression of cancers. It is imperative to fully understand their regulatory networks and explore their therapeutic potential in HCC. Bai *et al.* have found that MicroRNA-214-3p inhibits ATF4 in HCC cells HepG2 and Hep3B, accelerates ferroptosis, and can be used as a new therapeutic target or prognostic marker for HCC treatment (Bai et al., 2020). Similarly, ubiquitin-like modifier activating enzyme 1 (UBA1), which has been reported to participate in the development of HCC by modulating cell phenotypes and ferroptosis via the NRF2 pathway, is proposed to be a promising diagnostic and prognostic indicator for HCC (Shan et al., 2020). In addition, other non-coding RNAs such as the LncRNA GABPB1-AS1 may be key molecules that regulate the ferroptosis process of HCC cells HepG2 caused by erastin (Qi et al., 2019). Circular RNAs (circRNAs) are a new class of non-coding RNAs backspliced from pre-mRNAs (Memczak et al., 2013). Circular RNAs (circ) are usually dysregulated in human diseases, including cancers (Barrett and Salzman, 2016; Bolha et al., 2017; Chen et al., 2017; Bi et al., 2018; Su et al., 2020), and have been confirmed to be involved in various malignant behaviors of HCC (Wang et al., 2018). The circIL4R is abnormally overexpressed in HCC tissues and cells, and its knockdown prevents HCC cell tumorigenesis and accelerates ferroptosis. CircIL4R directly sponges microRNA-541-3p, and inhibition of miR-541-3p mitigated the effects of circIL4R knockdown on HCC cells; this suggests that circIL4R served as a tumor promoter and ferroptosis inhibitor in HCC by the miR-541-3p/GPX4 network (Xu et al., 2020). Recently, another circular RNA, cIARS, has been described as an important positive regulator of sorafenib-induced ferroptosis by suppressing the inhibition of autophagy mediated by the RNA binding protein ALKBH5 (Liu Z. et al., 2020).

In addition to miRNAs, RNA-binding proteins (RBP) were recently found to play roles in ferroptosis. For example, Qi Wang *et al.* have shown that the RNA-binding protein DAZAP1 could suppress ferroptosis in HCC cells HepG2, SMMC-7721, Hep3B, Bel-7402, Huh7 and L02 by interacting with the SLC7A11 mRNA to affect the sensitivity of HCC cells to sorafenib (Wang et al., 2021). Several other RBPs such as ZFP36 (Zhang et al., 2020b) IRP2 (Moroishi et al., 2011) have been reported to regulate ferroptosis. ELAV like RNA binding protein 1 (ELAVL1), which is highly expressed in many cancers, was a key target of ferroptosis induced by Erastin or Sorafenib in hepatic stellate cells. Up-regulation of ELAVL1 triggered by Erastin or Sorafenib promoted the production of BECN1/Beclin1 by binding to the AU-rich elements in the 3ʹ-UTR of BECN1 mRNA, thereby triggering autophagy activation, and ultimately promoting autophagic ferritin screening and ferroptosis (Zhang et al., 2018).

#### 3.2.2 ACSL4

Acyl-CoA synthetase long-chain family member 4 (ACSL4), a ferroptosis-positive activating enzyme that esterifies free PUFA and binds to membrane phospholipids by LPCAT3, is considered a ferroptosis biomarker in hepatocellular carcinoma and has been proposed to be useful to predict the sensitivity of Sorafenib in HCC (Feng et al., 2021). Furthermore, ACLS4 was found to have a differential expression profile in hepatocellular carcinoma and gastrointestinal hepatic metastases. Therefore, it could be used to differentiate HCC from other forms of liver cancer and indicate that up-regulation of fatty acid metabolism is a potential chemotherapeutic target for the treatment of HCC (Ndiaye et al., 2020).

#### 3.2.3 Metallothionein in Ferroptosis of HCC

Metallothionein (MT) is a family of small proteins widely expressed in eukaryotic cells. It is a low molecular weight protein and is highly rich in cysteine. It is highly induced in the reaction of different metal ions, cytokines, and free radicals and plays a critical role in detoxifying heavy metals and antioxidants (Coyle et al., 2002). Metallothionein-1G (MT-1G), a member of the MT family, was recently identified as a negative regulator of ferroptosis and a positive regulator of sorafenib resistance in HCC and could be used as a biomarker to explore the impact of Sorafenib on redox metabolism of cancer cells. Houessinon *et al.* found that HCC cells line Huh7 exposed to Sorafenib have enhanced expression of the metal ion protein-1 (MT1) gene due to the activity of the transcription factor NRF2, which has a binding site in an antioxidant response element found in the MT-1G promoter. The group also reported that sorafenib-treated patients have an increased level of MT1 protein, which was associated with reduced overall survival (Houessinon et al., 2016). Later, Sun *et al.* have sought to elucidate the mechanisms underlying the action of MT-1G on sorafenib resistance and discovered that MT-1G might facilitate sorafenib resistance by inhibiting ferroptosis (Sun et al., 2016).

## 4 Conclusion and Perspectives

Ferroptosis, characterized by iron and lipid peroxide-dependent cell death, has unique morphological and biological properties that have attracted widespread attention, as it can be induced in various cancers. Ferroptosis can be controlled by the key glutathione peroxidase 4 (Gpx4), antioxidants, and iron chelating agents. In addition to the conventional GSH-GPX4 axis, we have reviewed and summarized various mechanisms of GPX4 independent regulation of ferroptosis and highlighted their therapeutic potential in HCC. More aggressive phenotypes of HCC were associated with the activation of signaling pathways that regulate cell cycle progression and mutations in the TP53 gene in at least half of patients with HCC (Zucman-Rossi et al., 2015; Calderaro et al., 2019). However, whether this group of HCC patients could benefit from ferroptosis-induced therapy as reported in other cancers is unknown. Protein kinases such as mitogen-activated protein kinases whose activities are heavily impaired in HCC have also been suggested to have a role in ferroptosis in several disease models, although their implication for ferroptosis in HCC remained to be elucidated. Investigation to better understand in depth the contribution of these pathways to ferroptosis could ultimately open a new avenue to improve the outcomes of patients with HCC. Preventing chemotoxicity to healthy cells is a major concern in cancer treatment. Although inducing ferroptosis could be a reliable strategy for treating patients with HCC, emerging evidence also supports its role in the pathogenesis of other liver diseases (Wu et al., 2021; Chen et al., 2022). Therefore, an in-depth understanding of the regulatory mechanisms of ferroptosis in healthy cells versus HCC cells is required to selectively attack cancer cells while protecting all healthy tissues.
